# Induced pluripotent stem cells in cartilage tissue engineering: a literature review

**DOI:** 10.1042/BSR20232102

**Published:** 2024-05-10

**Authors:** Amani Y. Owaidah

**Affiliations:** Department of Clinical Laboratory Sciences, College of Applied Medical Sciences, Imam Abdulrahman Bin Faisal University, Dammam, Saudi Arabia

**Keywords:** 3D-bioprinting, Cartilage, Human induced pluripotent stem cells, osteoarthritis, stem cells

## Abstract

Osteoarthritis (OA) is a long-term, persistent joint disorder characterized by bone and cartilage degradation, resulting in tightness, pain, and restricted movement. Current attempts in cartilage regeneration are cell-based therapies using stem cells. Multipotent stem cells, such as mesenchymal stem cells (MSCs), and pluripotent stem cells, such as embryonic stem cells (ESCs), have been used to regenerate cartilage. However, since the discovery of human-induced pluripotent stem cells (hiPSCs) in 2007, it was seen as a potential source for regenerative chondrogenic therapy as it overcomes the ethical issues surrounding the use of ESCs and the immunological and differentiation limitations of MSCs. This literature review focuses on chondrogenic differentiation and 3D bioprinting technologies using hiPSCS, suggesting them as a viable source for successful tissue engineering. Methods: A literature search was conducted using scientific search engines, PubMed, MEDLINE, and Google Scholar databases with the terms ‘Cartilage tissue engineering’ and ‘stem cells’ to retrieve published literature on chondrogenic differentiation and tissue engineering using MSCs, ESCs, and hiPSCs. Results: hiPSCs may provide an effective and autologous treatment for focal chondral lesions, though further research is needed to explore the potential of such technologies. Conclusions: This review has provided a comprehensive overview of these technologies and the potential applications for hiPSCs in regenerative medicine.

## Introduction

Cartilage is an avascular, aneural, and alymphatic white connective tissue covering the end of long bones. Once damaged, it lacks intrinsic healing ability due to its avascular nature [1,2]. Articular cartilage (AC) provides wear resistance and frictionless, lubricated, and compressible surfaces for joint movement [3,4]. AC comprises chondrocytes and an extracellular matrix (ECM), which includes water, proteoglycans, and collagen [5] Osteoarthritis (OA) is a degenerative disease that results from AC injury, leading to severe joint deformity, stiffness, and limited mobility [6,7]. However, OA is now widely accepted as a whole-joint disease in which several tissues, including synovium, menisci, ligaments, fat pads, and tendons, play key roles [8]. According to World Health Organization (WHO), by 2023, OA is expected to be the fourth leading cause of disability [9]. Finding ways to regenerate AC and restore normal joint function is essential to treat and improve the quality of life of those suffering from OA.

Cartilage destruction occurs as chondrocytes produce proteolytic enzymes that degrade collagen and proteoglycans. Factors such as age, genetics, metabolic syndrome, trauma, injury, and stress play a role in the progression of OA [10]. In the United States, OA has been reported to affect approximately 10–15% of people over 60 [11]. A severe case of OA may require knee replacement in addition to pharmacological and non-pharmacological treatments [12]. Cartilage matrix homeostasis is essential as chondrocytes respond to cytokines, stimuli, and growth factors [13]. An imbalance in homeostasis leads to the degradation of ECM, resulting in OA [14]. Modifications brought about by OA directly influence the biomechanical responses of various joint tissues, particularly the AC. Where they are critical in bearing biomechanical strain due to their load-bearing surfaces that offer minimal friction and wear resistance and exhibit gliding characteristics. The physiological changes induced by OA also affect chondrocytes and chondrons, which alter their biomechanical nature. Interestingly, in comparison with healthy chondrocytes, OA chondrocytes have a lower elastic modulus and viscosity [15,16].

Depending on the type and size of the affected area, various treatments are available for OA. Pharmaceutical treatments aim only to reduce pain and not to restore cartilage damage [17,18]. In addition to pharmaceutical pain management, surgical therapies such as arthroscopy, arthroplasty, microfracture, mosaicplasty, and autologous chondrocyte implantation (ACI) can restore damaged tissue [2,19]. However, surgical therapies are invasive and carry risks, such as infection, bleeding, and nerve damage, and also the recovery from surgery is lengthy with extensive physical therapy [20]. ACI is the only proven therapy that can temporarily restore normal function in people with cartilage degeneration and delay the need for joint replacement surgery. Unfortunately, this technique cannot effectively treat the widespread cartilage damage that occurs in OA [21]. New approaches, such as cell-based therapies using mesenchymal stromal cells (MSCs) isolated from adipose tissue, bone marrow, synovium fluid, or umbilical cord, have shown promising results in cartilage regeneration in OA [22]. OA-MSCs have been successfully used to produce 3D cartilage tissue since 2007 [23]. Due to their chondrogenic potential, preclinical and clinical studies have investigated several treatments for OA that involve the introduction of MSCs into damaged cartilage regions [21,24]. However, the low efficiency of MSCs in differentiation, researchers have investigated the use of embryonic stem cell-derived mesenchymal stem cells (ES-MSCs) as therapy for various disorders [25,26]. Multiple injections of ES-MSCs have been shown to improve OA symptoms in rat models, and ES-MSCs could be an effective, potentially endless cellular source for OA treatment [22].

Since the introduction of induced pluripotent stem cells (iPSCs) by the Yamanaka group in 2006 has revolutionized regenerative medicine and created incredible opportunities, as these cells exhibit properties similar to ESCs overcoming the ethical issues surrounding the use of embryonic material [2,27,28]. In the future, iPSCs could treat cartilage defects in clinical settings [29]. Recent studies have demonstrated a variety of protocols for chondrogenic differentiation, such as high-density mass, micro mass, monolayer culture, embryoid body formation, and directed differentiation methods [2]. To help treat OA, new technologies such as hiPSCs and 3D bioprinting are being developed and explored [28]. This review discusses the various methods employed in chondrogenic differentiation and the recent trends of 3D bioprinting used to regenerate cartilage which could be a possible approach for treating OA.

### Methodology

The present review article focused on attaining knowledge on chondrogenic differentiation using hiPSCs and its recent trends in 3D printing technology. A literature search was conducted by two separate reviewers using scientific search engines like PubMed, MEDLINE, and Google Scholar databases with the terms ‘cartilage tissue engineering,’ ‘osteoarthritis treatment,’ ‘hiPSCs,’ and ‘3D bioprinting’ to retrieve published literature on chondrogenic differentiation and their different protocols. The inclusion criteria for the current literature review were studies published in English, studies on chondrogenic differentiation, and tissue engineering using hiPSCs, with *in vitro* and *in vivo* models

This review discusses the current cell-based therapies for cartilage tissue engineering, chondrogenic differentiation protocols, 3D bioprinting also offers promising treatment options for OA. This article highlights the potential of iPSCs in regenerative medicine, emphasizing the need for continued research and innovation to improve outcomes for patients with OA.

### Chondrogenic differentiation of hiPSCs

An exciting new approach to cartilage tissue engineering involves chondrogenic differentiation of hiPSCs. As a result of cartilage tissue formation and maintenance, chrondrocytes play a vital role. Repairing cartilage defects using chondrocytes is challenging, as they have limited self-renewal and tissue regeneration capacity; here hiPSCs take advantage of these challenges [30]. In this context, hiPSCs are valuable alternatives, as they can differentiate into chondrocytes and produce cartilage [13,31]. Different methods can be used to induce cartilage differentiation in hiPSCs, including the use of growth factors such as transforming growth factor-β (TGF-β), bone morphogenetic protein (BMP), and fibroblast growth factor (FGF) [32]. Several studies have reported successful chondrogenic differentiation of hiPSCs *in vitro* and *in vivo*, using different approaches. Growth factors activate specific signaling pathways that promote the expression of chondrogenic markers, including *SOX9* (SRY (sex determining region Y-box 9), *COL2A1* (Collagen type 2), and *ACAN* (AGGRECAN), these markers can be validated under *in vitro* conditions in induced hiPSC cell lines [33]. Differentiating hiPSCs into functional cartilage tissue can also be improved using mechanical stimulation and 3D culture systems [7]. For example, a study by Ko et al. demonstrated that hiPSC-derived chondrocytes could produce cartilage-like tissue in a mouse model of OA [34]. Similarly, another study by Toh et al. showed that hiPSC-derived chondrocytes could repair cartilage defects in a rat model of OA [35]. These findings suggest that the chondrogenic differentiation of hiPSCs holds great promise for cartilage tissue engineering and regenerative medicine.

Differentiation protocols must consider several important factors, such as growth factor concentration, cell density in culture, type of cell population, and biomaterials that mimic the *in vivo* microenvironment [36]. Some of the protocols used to differentiate iPSCs into chondrocyte-like cells include the following:

1. Embryoid bodies (EBs) formation
2. Coculture
3. Direct differentiation [7]

To evaluate successful chondrogenic differentiation, certain markers should be considered. Table 1 lists the chondrogenic differentiation markers used to assess chondrogenic differentiation.

**Table 1:** List of chondrogenic markers

Chondrogenic markers	Description
COL1A1	Gene responsible for type I collagen expression in fibrochondrocytes
COL2A1	Gene responsible for type II collagen, the primary component of articular cartilage
COL10A1	Gene responsible for collagen X, which is expressed primarily by hypertrophic chondrocytes
ACAN	Aggrecan, the abundant proteoglycan in articular cartilage
SOX9	SRY-like-box protein 9 plays a role in collagen II and aggrecan activation
COMP	Chondrocytes predominantly express a non-collagen protein

Abbreviations: ACAN, aggrecan; COMP, cartilage oligomeric matrix protein; COL1A1, collagen type 1, α1; COL2A1, collagen type 2, α1; COL10A1, collagen type 10, α1; SOX9, SRY-box 9; SRY, sex determining region Y.

#### Embryoid bodies (EB) formation

One of the most established stem cell differentiation protocols involves the formation of EBs. In addition, hiPSCs can also be differentiated into EBs with respect to OA differentiation. As hiPSCs aggregate spontaneously, the resulting structures resemble early embryos in three dimensions [37]. EBs are aggregates of 3D cells that reflect embryonic differentiation into the three germ layers (endoderm, mesoderm, and ectoderm) [38] (Table 2). EBs are obtained by culturing iPSCs in ultra-low attachment flasks to prevent surface attachment and promote cell aggregation [39]. Without exogenous growth factors, EBs can differentiate into chondrocytes, thus providing an added advantage compared with other chondrogenic differentiation methods [38]. Chondrogenic differentiation of hiPSCs into cartilage involves two critical steps: induction of mesenchymal-like outgrowth cells and accumulation of chondrogenic pellets [34,40]. According to Rim et al. hiPSCs can be induced in EBs that can differentiate into chondrocytes that express chondrogenic markers, such as SOX9 and COL2A1 [41]. Costa et al. used TGF-3 and BMP-6 to induce chondrogenesis in hiPSC-derived EB and showed that the resulting chondrocytes produced cartilage tissue *in vitro* [42]. Kim et al. successfully used EB formation as the first protocol for chondrogenesis [43]. In the EB protocol, EBs are cultured in a standard chondrogenic medium supplemented with specific growth factors that induce chondrogenic differentiation, such as TGF-β1, TGF-β3, BMP-2, BMP-4, BMP-6, and BMP-7 [36]. Auguyniak et al. used hiPSCs to generate EBs cultured in a chondrogenic medium containing TGF-β1, BMP-2, and insulin-like growth factor-1 (IGF-1) and observed a promising outcome [44] Rim et al. used TGF-β1, BMP-2, and dexamethasone to induce chondrogenic differentiation of hiPSC-derived Ebs [41]. Compared with primary human chondrocytes, chondrocytes derived from this method had similar gene expression profiles (Table 2).

**Table 2:** Studies using embryoid body formation, coculturing, and directed differentiation for chondrogenic differentiation

Differentiation type	Differentiation duration	Pellet culture	Differentiation medium	Outcome gene expression of chondrogenic markers (relative expression to undifferentiated cells)		Limitations	Reference
Embryoid body formation	35 days	+	CDM No supplements	**SOX9, *P*<0.01**	**COL10A1 NT**	Osteogenic Markers Runx2, osteocalcin was up-regulated *P*<0.01	[54]
				**COL2A1, *P*<0.01**	**COL1A1 NT**		
				**ACAN NT**			
	35 days	+	CDM +TGF-β3	**SOX9, *P*<0.01**	**COL10A1 NS**		[34]
				**COL2A1, *P*<0.01**	**COL1A1 NS**		
				**ACAN, *P*<0.01**			
	38 days	+	CDM + TGF-β1 Cells were sorted for CD73 and CD105 before differentiation	**SOX9, *P*<0.05**	**COL10A1, *P*<0.05**	Fibrocartilage marker COL1A1 was not tested, and *in vivo* testing demonstrated progression toward the bone	[48]
				**COL2A1, *P*<0.05**	**COL1A1, NS**		
				**ACAN, *P*<0.05**			
	45 days	+	CDM +TGF-β3+BMP-2	**SOX9, *P*<0.01**	**COL10A1, NT**	sGAG level was limited to qualitative analysis through toluidine blue staining. Low cell count of the generated chondrocytes	[40]
				**COL2A1, NS**	**COL1A1, *P*<0.05**		
				**ACAN, NT**			
	28 days	-	CDM +TGF-β3	**SOX9, *P*<0.001**	**COL10A1, NT**	sGAG level was not determined Gene expression of fibrocartilage marker COL1A1 and hypertrophic chondrocytes COL10A1 was not tested. The scale-up possibility has not been tested	[44]
				**COL2A1, NT**	**COL1A1, NT**		
				**ACAN, NT**			
Coculture	14 days	+	Primary chondrocytes The iPSC cultured with TGF- β1	**SOX9, NT**	**COL10A1, NT**	Exposure to undefined factors from cocultured cells. Gene expression of fibrocartilage marker COL1A1 and hypertrophic chondrocytes COL10A1 was not tested	[3]
				**COL2A1, *P*<0.05**	**COL1A1, NT**		
				**ACAN, *P*<0.05**			
	51 days	+	Primary chondrocytes	**SOX9, NS**	**COL10A1, NT**	De-differentiation of generated cells in monolayer. Pellet cultures analysis was limited to qualitative analysis	[49]
				**COL2A1, NS**	**COL1A1, up-regulated**		
				**ACAN, NS**			
				**COL2A1, NT**	**COL1A1, NT**		
				**ACAN, NT**			
Directed differentiation	14 days	-	Oldershaw et al., 2010 chemically defined the method	**SOX9, *P*<0.01**	**COL10A1, NT**	Gene expression of fibrocartilage marker COL1A1 and hypertrophic chondrocytes COL10A1 was not tested. The scale-up possibility has not been tested	[56]
				**COL2A1, *P*<0.01**	**COL1A1, NT**		
				**ACAN, NT**			
	15 days	+	CDM +TGF-β3+BMP-2	**SOX9, *P*<0.05**	**COL10A1, NT**	Gene expression of fibrocartilage marker COL1A1 and hypertrophic chondrocytes COL10A1 were not tested. Scale-up possibility has not been tested	[54]
				**COL2A1, *P*<0.05**	**COL1A1, NT**		
				**ACAN, NT**			
	15days	+	CDM +BMP-2	**SOX9, *P*<0.05**	**COL10A1, NT**	Gene expression of fibrocartilage marker COL1A1 and hypertrophic chondrocytes COL10A1 were not tested. Scale-up possibility has not been tested	[57]
				**COL2A1, *P*<0.05**	**COL1A1, NT**		
				**ACAN, *P*<0.05**			
	14 days	+	Oldershaw et al., 2010 chemically defined the method	**SOX9, *P*<0.001**	**COL10A1, NT**	Gene expression of fibrocartilage marker COL1A1 and hypertrophic chondrocytes COL10A1 were not tested Scale-up possibility has not been tested. The hyaline cartilage marker was not significantly up-regulated	[50]
				**COL2A1, NS**	**COL1A1, NT**		
				**ACAN, *P*<0.001**			
	14 days	+	Oldershaw et al., 2010 chemically defined the method	**SOX9, *P*<0.0001**	**COL10A1 *NT***	Gene expression of fibrocartilage marker COL1A1 and hypertrophic chondrocytes COL10A1 were not tested. Scale-up possibility has not been tested	[33]
				**COL2A1, *P*<0.0001**	**COL1A1, NT**		
				**ACAN, *P*<0.0001**			
	42 days	-	CDM+ TGF-β3	**SOX9, *P*<0.001**	**COL10A1, NT**	Pellet cultures showed signs of hypertrophy	[53]
				**COL2A1, *P*<0.001**	**COL1A1, NT**		
				**ACAN, *P*<0.001**			

Abbreviations: CDM, chondrogenic differentiation medium; NS, not significant; NT, not tested.

Lee et al. reported that a cocktail of defined growth factors allowed more efficient differentiation of hiPSCs into chondrocytes in fibronectin-coated plates within 14 days using EB [45]. Despite this advantage, this protocol requires many growth factors at high concentrations (up to 100 ng/ml), making it expensive and limiting its potential clinical application [45]. Despite its advantages, such as its ability to mimic early embryonic development and promote cellular interactions, the use of EBs for chondrogenic differentiation has some limitations, which mainly include heterogeneity, little control over the differentiation process, time-consuming, and risk for teratoma formation [45–47] (Table 2). Li et al. successfully generated hiPSCs (including EB formation) in 21 days and noticed high expression levels of chondrogenic markers like *COL2, COL10, AGGRECAN,* and *COL9* [48].

#### Coculturing protocol

Another method is coculturing hiPSCs with chondrocytes. This procedure creates a microenvironment that promotes chondrogenic differentiation by growing hiPSCs alongside chondrocytes (Table 2). Furthermore, coculture systems mimic the *in vivo* stimuli environment of cartilage tissue, making them more physiologically relevant for chondrogenic differentiation. Wei et al. used a two-step protocol where EB formation was cocultured with primary human chondrocytes in a chondrogenic medium with alginate for 28 days [3]. The differentiated cells exhibited chondrocyte-like morphology and expressed AGGRECAN and COL-II. Biochemical assays showed that differentiated cells produced components of the extracellular matrix typical of hyaline cartilage, including glycosaminoglycan and collagen. A study by Qu et al. used the coculture method supplemented with dexamethasone, L-ascorbic acid 2-phosphate, proline, pyruvate, and TGF-β3 [49]. Cells were cultured for 21 days and observed up-regulation of chondrogenic markers, including collagen type II and AGGRECAN, and down-regulation of markers associated with undifferentiated cells, such as OCT4 and SOX2. The alginate matrix was a suitable scaffold for the chondrogenic differentiation of iPSC. The results suggest that this approach could be further developed to produce functional cartilage tissue for clinical applications [3]. Table 2 highlights the articles that used coculturing protocol in the chondrogenic differentiation.

#### Directed differentiation

Directed differentiation of human-induced pluripotent stem cells (hiPSCs) into chondrocytes is a promising approach to cartilage tissue engineering as it bypasses the stage of MSCs [50]. Oldershaw et al. initially described a method to direct human embryonic stem cells (hESCs) to differentiate into chondrocytes, which form and maintain cartilage tissue: a step-by-step protocol-induced mesoderm formation, chondrogenic differentiation, and chondrocyte maturation [51]. As a result, chondrocytes produced cartilage-like tissue *in vitro* and expressed chondrogenic markers (SOX9, ACAN, and COL2A1). This approach offers a more controlled and efficient method for producing chondrocytes for cartilage tissue engineering and regenerative medicine than other methods of chondrogenic differentiation. Based on this technology, protocols for iPSCs were developed to induce chondrogenesis by chemical definition. Table 2 provides an overview of the research using directed differentiation methods. Later, Toh et al. used TGF-β3 and BMP-6 to induce chondrogenesis in hiPSCs and showed that the resulting chondrocytes could repair cartilage defects in a rat model of osteoarthritis [35]. Another study by Nakagawa et al. used a combination of TGF-β1 and BMP-2 to induce chondrogenic differentiation of hiPSCs and demonstrated the successful formation of hyaline cartilage *in vitro* [52].

Direct differentiation of hiPSCs into chondrocytes, without contamination-prone coculture systems, is possible. Nejadnik et al. reported a method to differentiate iPSCs into chondrocytes in approximately 42 days using a chondrogenic differentiation medium and TGF-β3 [53]. Similarly, Matsumoto et al. reported a successful protocol with a chondrogenic differentiation medium and TGF-β3 and BMP-2 [54]. In another study by Saito et al. suggested that they induced chondrogenic differentiation of hiPSC and formed hyaline cartilage tissue when implanted subcutaneously into immunodeficient mice [33]. However, the study highlighted the risk of tumorigenesis due to the formation of teratomas in the implanted tissue. The directed differentiation method is chemically defined, making it more reproducible than other differentiation methods. Also, coculture with ESCs provides a more natural and physiological environment, producing more homogeneous chondrogenic cells [55]. Several reports also suggested the high levels of gene expression of COL2A1, SOX-9, and ACAN in 14-15 days of differentiation [54,56,57].

### 3D cartilage tissue engineering using hiPSC

Regenerative medicine uses tissue engineering to develop functional tissues or organs that can replace or repair damaged tissues or organs. This field involves the creation of 3D structures that mimic the structure and function of native tissues and organs using cells, biomaterials, and biochemical factors. It was reported that hiPSCs are an effective approach for repairing damaged cartilage and restoring joint functions [2]. Compared with other cell sources, hiPSCs can differentiate into chondrocytes, the main cells responsible for cartilage formation and maintenance [58].

In 3D bioprinting, the two main approaches are scaffold-based and scaffold-free are used to create tissue constructs [59]. Using hiPSCs, a scaffold-based system can engineer 3D cartilage tissue. As a result of the scaffold, hiPSCs can grow and organize into functional cartilage tissues [60]. A scaffold provides mechanical support, cell guidance, and a platform for delivering biochemical factors that promote tissue growth [61]. In contrast, scaffold-free tissue engineering does not require the use of scaffolds, and instead, they rely on cell self-assembly and organization. This approach uses cell sheet engineering and spheroid and organoid cultures to create 3D structures without scaffolds [61].

Scaffold-based tissue engineering presents multiple benefits compared with scaffold-free methods. One key advantage is the ability to manage the framework and mechanical characteristics of the scaffold, which can be customized to align with the features of the original tissue. This enables the creation of tissue-engineered constructs that accurately imitate the structure and functionality of native tissues [62]. In addition, they facilitate the delivery of biochemical elements to cells, which encourages tissue development and differentiation. This is especially valuable for tissues that require complex microenvironments, such as bone or cartilage, which require the delivery of growth factors and other signaling molecules to foster tissue generation [63]. However, scaffold-free tissue engineering can produce 3D structures that closely resemble native tissues without requiring a scaffold. This enables the creation of tissue-engineered constructs that closely mimic the architecture and functionality of natural tissue.

Additionally, scaffold-free tissue engineering eliminates the possibility of immune rejection because it does not incorporate foreign materials that may be identified by the host’s immune system [64]. Scaffold-based and scaffold-free methods present unique advantages and disadvantages, with the chosen technique relying on the specific tissue or organ being engineered and its intended clinical use [65]. Further investigation is required to enhance both methodologies and to devise novel tissue engineering strategies to address the limitations of the field.

Bioprinting is an interdisciplinary field that combines the strengths and advantages of 3D printing and regenerative medicine to bridge the gap between biology and engineering. Using traditional additive manufacturing techniques, bioprinting technology creates microscale tissues by precisely deploying bioinks containing cells [66]. Bioprinting has garnered interest in hiPSCs because of their capacity to generate intricate 3D formations that resemble the architecture and functionality of natural tissues or organs [67]. 3D bioprinting is a burgeoning technology that presents a promising method for tissue engineering using hiPSCs, where hydrogels, ECM proteins, and synthetic polymers are used [68]. Employing hiPSCs in 3D bioprinting provides numerous benefits compared with other cell sources, such as their ability to transform into various cell types and their potential for limitless growth and accessibility [69]. Multiple studies have documented the successful bioprinting of hiPSCs for tissue engineering. Extrusion-based, Inkjet, and laser-assisted techniques are used in 3D bioprinting [70,71].

Nguyen and colleagues employed a 3D bioprinting technique to create iPSCs with a composite bio-ink made of nanofibrillated cellulose and alginate (NFC/ALG) combined with irradiated human chondrocytes. After 5 weeks, they observed the formation of hyaline-like cartilaginous tissue, accompanied by an increased presence of chondrocytes within the bioprinted structures. These findings imply that the optimal bioink for 3D bioprinting of iPSCs, and their direct conversion to chondrocytes, may offer a novel regenerative treatment for damaged cartilage [72]. A bioink was created by mixing COL type I and agarose (AG) with sALG in 2018, which embedded chondrocytes to 3D print cartilage tissue *in vitro*, which enabled higher mechanical strength [73]. Hontani et al. developed a unique approach to chondrogenic differentiation, which involves using ultra-purified alginate gel as a 3D scaffold and gradually transforming iPSCs into chondrocytes through the MSC-like cell phase. As iPSC-MSCs were cultivated, SOX9, Col2A1, and aggrecan expression increased significantly [74]. In another study, Bioink based on nanofibrillated cellulose (NFC) composite was used for 3D bioprinting methods to fabricate cartilage tissue structures from iPSCs with irradiated human chondrocytes (iCHons) [75]. The hyaline-like cartilage tissue maintained pluripotency for five weeks, as confirmed by the expression of collagen type II and the tumor-causing gene OCT4 [54]. Nakagawa et al. used a combination of TGF-β1 and BMP-2 to initiate the chondrogenic differentiation of hiPSCs and successfully formed hyaline cartilage *in vitro* [52].

**Figure 1:**
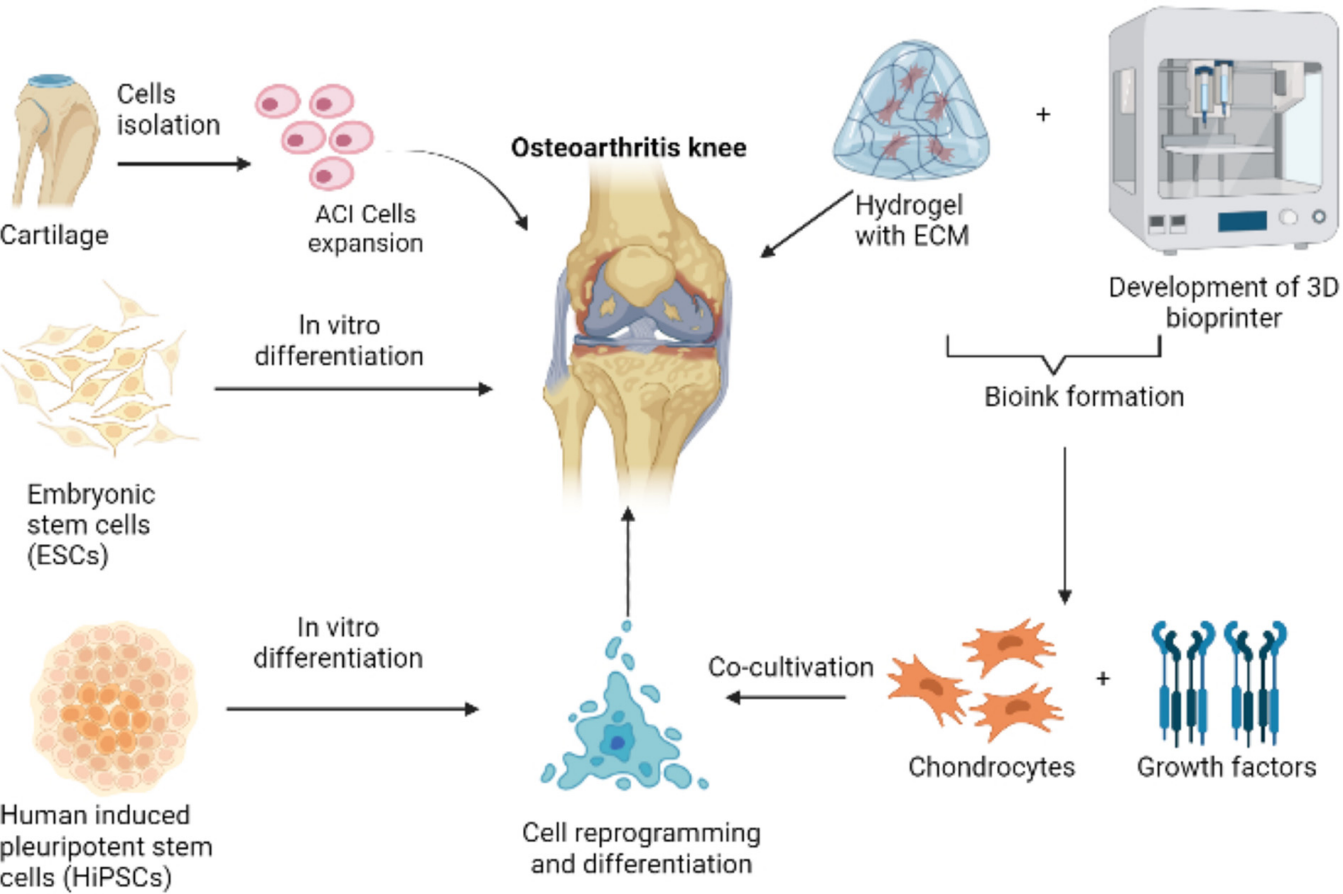
A schematic representation of cell-based therapies and 3D bioprinting practice in osteoarthritis

Furthermore, the study discovered that incorporating a poly(lactic-co-glycolic acid) (PLGA) scaffold enhanced the chondrogenic differentiation of hiPSCs and the development of functional cartilage tissue. Figure 1 illustrates the combined approach to OA repair using cell-based therapies and 3D bioprinting technology. iPSCs are derived from adult somatic cells to avoid the ethical issues of using ESCs. Furthermore, they can be derived from tissue sources, such as fibroblasts, and do not require invasive methods such as bone marrow or adipose tissue biopsies [75]. The NFC/ALG composite ink formulation has been brought to market under the brand name CELLINK®. It has undergone *in vitro* and *in vivo* testing with human chondrocytes and iPSC cells originating from chondrocytes [76].

Lee et al. conducted a study using a 3D bioprinting technique to create a customized cardiac tissue structure by incorporating hiPSC-derived cardiomyocytes and a hydrogel predominantly composed of collagen. Using a rat model of myocardial infarction, the engineered cardiac tissue was shown to demonstrate characteristics similar to native cardiac tissue and to improve cardiac function [77]. Similarly, Zhang et al. employed a 3D bioprinting approach to producing a cartilage tissue structure using hiPSCs and a hydrogel mainly composed of silk fibroin. The study concluded that the fabricated cartilage tissue exhibited properties similar to those of natural cartilage tissue and could correct cartilage defects in a rabbit model of OA [78]. Additionally, the study demonstrated the feasibility of using magnetic nanoparticles to enhance the targeting and retention of hiPSCs in damaged cartilage tissue [78]. Table 3 lists some articles in which different 3D printing technologies have been successfully used to achieve optimal results in chondrogenesis. In summary, 3D bioprinting with hiPSCs holds great promise as a strategy for treating OA and regenerating a damaged cartilage tissue. However, further research is necessary to optimize 3D bioprinting and develop new techniques to fabricate complex tissues or organs utilizing hiPSCs.

**Table 3:** Summary of studies on 3D printing technology used in cartilage repair

Bio-ink	GF	Technique	Cells	Outcome	Reference
CS	TGF- β3 and BMP-3	CTE	human chondrocytes 7.5 × 10^5^ cells/ml	**✓** Cartilage-like tissue formation in 4 weeks of culture **✓** Up-regulation of COL2, ACAN, SOX9 genes	[79]
Gel/HA/glycerol	TGF-β and BMP-4	CTE	Rabbit BMSC	**✓** Good mechanical support **✓** Enhanced cell viability and proliferation **✓** High expression of COL2A1 and PRG4 (proteoglycan 4) **✓** Detection of osteogenic markers	[80]
NFC/ALG	–	Extrusion-based	Human chondrocytes and iPSC cells	**✓** Increased mechanical stiffness and stability	[76]
NFC/ALG	–	Extrusion-based	HiPSCs cocultured with human chondrocytes	**✓** Better cell proliferation **✓** Good pluripotency **✓** Enhanced chondrogenic phenotype expression **✓** High expression of COL2 SOX9 and ACAN	[72]
Type 1 COL	–	Extrusion-based	Rat chondrocytes	**✓** Good stability **✓** Good printability **✓** Expression of COL2, ACAN and SOX9 **✓** Promising biological functionality **✓** GAG accumulation	[73]
GelMA/pluronic	–	Inkjet, PCL, and extrusion-based	Porcine BMSCs cocultured with chondrocytes	**✓** High integration **✓** Mechanical support **✓** Enhanced osteochondral pathways	[81]
Carboxymethyl CS	–	Extrusion-based	Rabbit chondrocytes (1 × 10^5^ cells/ml)	**✓** Good stability **✓** Low cytotoxicity **✓** High expression of SOX9 **✓** Good cell proliferation **✓** Fast gelation **✓** High precision during printing	[82]
CS	–	Extrusion-based	Mouse chondrogenic cell line (ATDC5, 10^6^ cells/ml)	**✓** High cell adhesion **✓** High biocompatibility **✓** Improved mechanical behaviour **✓** High cell growth **✓** Enhanced expression of chondrogenic markers like COL1, COL2, and ACAN	[83]
Cartilage bio-ink	–	Extrusion-based	Rabbit chondrocytes	**✓** Mechanical support **✓** Inhibits chondrocyte hypertrophy	[84]
Nano-HAP/type 1 COL	–	Laser-based	Mouse chondrocytes	**✓** High viability and proliferation **✓** Bone regeneration	[85]

Abbreviations: ALG, alginate; CS, chitosan; CTE, cartilage tissue engineering; GAG, glycosaminoglycan; Gel, gelatine; GF, growth factor; HA, hyaluronic acid; HAP, hydroxyapatite; NFC, nanofibrillated cellulose.

The results of this review suggest that hiPSCs can regenerate cartilage successfully. However, further research is required to address the potential risk of tumorigenesis associated with hiPSCs. Hence, other studies are needed to address potential risks associated with hiPSCs and ensure their safety. Moreover, *in vivo* studies could assess the long-term safety and efficacy of hiPSC-derived cartilage and the molecular and genetic changes occurring during iPSC generation and differentiation.

## Conclusion

The ability of stem cells to differentiate into chondrocytes, the cells responsible for forming, and maintaining cartilage, is one of the major advantages of hiPSCs for cartilage tissue engineering. Further, hiPSCs combined with bioprinting technology could lead to a revolution in cartilage tissue engineering. Using bioprinting, complex 3D structures mimicking the native tissue can be designed with precise placement of cells and biomaterials. From hiPSCs, cartilage tissue can be created with greater accuracy, reproducibility, and efficiency using bioprinting than traditional methods. In addition, custom scaffolds made according to the geometry of the defect site might improve cartilage regeneration and repair outcomes. HiPSCs combined with bioprinting could overcome many limitations of the current cartilage tissue engineering strategies and provide an alternative to treat cartilage defects and osteoarthritis. However, other innovative bioinks, rich in chondrogenic cells and growth factors, are still needed to optimize cartilage tissue engineering strategy and promote cartilage repair. However, this technology offers a new frontier for bio-fabrication, demonstrating the potential for revolutionizing 3D bioprinting.

## Abbreviations

ACI: autologous chondrocyte implantation
BMP: bone morphogenetic protein
CDM: chondrogenic differentiation medium
CTE: cartilage tissue engineering
EB: embryoid body
ES-MSC: embryonic stem cell-derived mesenchymal stem cell s
ESC: embryonic stem cells
FGF: fibroblast growth factor
GAG: glycosaminoglycan
hESC: human embryonic stem cells
hiPSC: human-induced pluripotent stem cells
MSC: mesenchymal stem cells
NFC: nanofibrillated cellulose
OA: osteoarthritis
TGF-β: transforming growth factor-β

## References

[1] Sophia Fox A.J., Bedi A. and Rodeo S.A. (2009) The basic science of articular cartilage: structure, composition, and function. Sports Health 1, 461–468 10.1177/1941738109350438 23015907 PMC3445147

[2] Lach M.S., Rosochowicz M.A., Richter M., Jagiełło I., Suchorska W.M. and Trzeciak T. (2022) The induced pluripotent stem cells in articular cartilage regeneration and disease modelling: are we ready for their clinical use? Cells 11, 529 10.3390/cells11030529 35159338 PMC8834349

[3] Wei Y., Zeng W., Wan R., Wang J., Zhou Q., Qiu S. et al. (2012) Chondrogenic differentiation of induced pluripotent stem cells from osteoarthritic chondrocytes in alginate matrix. Eur. Cell Mater. 23, 1–12 10.22203/eCM.v023a01 22241609 PMC7447074

[4] Evenbratt H., Andreasson L., Bicknell V., Brittberg M., Mobini R. and Simonsson S. (2022) Insights into the present and future of cartilage regeneration and joint repair. Cell Regen 11, 3 10.1186/s13619-021-00104-5 35106664 PMC8807792

[5] Mow V.C., Tohyama H. and Grelsamer R.P. (1994) Structure-function of knee articular cartilage. Sports Med. Arthrosc Rev. 2, 189–202 10.1097/00132585-199400230-00003

[6] Hunziker E.B. (2002) Articular cartilage repair: basic science and clinical progress. A review of the current status and prospects. Osteoarthritis Cartilage 10, 432–463 10.1053/joca.2002.0801 12056848

[7] Liu H., Yang L., Yu F.F., Wang S., Wu C., Qu C. et al. (2017) The potential of induced pluripotent stem cells as a tool to study skeletal dysplasias and cartilage-related pathologic conditions. Osteoarthritis Cartilage 25, 616–624 10.1016/j.joca.2016.11.015 27919783

[8] Shi X., Mai Y., Fang X., Wang Z., Xue S., Chen H. et al. (2023) Bone marrow lesions in osteoarthritis: from basic science to clinical implications. Bone Rep. 18, 101667 10.1016/j.bonr.2023.101667 36909666 PMC9996250

[9] Paul C. (2004) The burden of musculoskeletal conditions at the start of the new millennium. Report of a WHO Scientific Group. Geneva: WHO Technical Report Series, 919, 2003, pp. 218. ISBN: 92-4-120919-4. Int. J. Epidemiol. 34, 228–229 10.1093/ije/dyh383 14679827

[10] Blagojevic M., Jinks C., Jeffery A. and Jordan K.P. (2010) Risk factors for onset of osteoarthritis of the knee in older adults: a systematic review and meta-analysis. Osteoarthritis Cartilage 18, 24–33 10.1016/j.joca.2009.08.010 19751691

[11] Zhang Y. and Jordan J.M. (2010) Epidemiology of osteoarthritis. Clin. Geriatr. Med. 26, 355–369 10.1016/j.cger.2010.03.001 20699159 PMC2920533

[12] Robinson W.H., Lepus C.M., Wang Q., Raghu H., Mao R., Lindstrom T.M. et al. (2016) Low-grade inflammation as a key mediator of the pathogenesis of osteoarthritis. Nat. Rev. Rheumatol. 12, 580–592 10.1038/nrrheum.2016.136 27539668 PMC5500215

[13] Liu Y., Shah K.M. and Luo J. (2021) Strategies for articular cartilage repair and regeneration. Front Bioeng. Biotechnol. 9, 770655 10.3389/fbioe.2021.770655 34976967 PMC8719005

[14] Brandt K.D., Dieppe P. and Radin E.L. (2008) Etiopathogenesis of osteoarthritis. Rheum. Dis. Clin. N. Am. 34, 531–559 10.1016/j.rdc.2008.05.011 18687271

[15] Belluzzi E., Todros S., Pozzuoli A., Ruggieri P., Carniel E.L. and Berardo A. (2023) Human cartilage biomechanics: experimental and theoretical approaches towards the identification of mechanical properties in healthy and osteoarthritic conditions. Processes 11, 1014 10.3390/pr11041014

[16] Pettenuzzo S., Arduino A., Belluzzi E., Pozzuoli A., Fontanella C.G., Ruggieri P. et al. (2023) Biomechanics of chondrocytes and chondrons in healthy conditions and osteoarthritis: a review of the mechanical characterisations at the microscale. Biomedicines 11, 1942 10.3390/biomedicines11071942 37509581 PMC10377681

[17] Chen T., Weng W., Liu Y., Aspera-Werz R.H., Nüssler A.K. and Xu J. (2021) Update on novel non-operative treatment for osteoarthritis: current status and future trends. Front Pharmacol. 12, 755230 10.3389/fphar.2021.755230 34603064 PMC8481638

[18] Grässel S. and Muschter D. (2020) Recent advances in the treatment of osteoarthritis. F1000Res 9, F1000, Faculty Rev-325 10.12688/f1000research.22115.1 32419923 PMC7199286

[19] Francis S.L., Di Bella C., Wallace G.G. and Choong P.F.M. (2018) Cartilage tissue engineering using stem cells and bioprinting technology-barriers to clinical translation. Front Surg. 5, 70 10.3389/fsurg.2018.00070 30547034 PMC6278684

[20] Gracitelli G.C., Moraes V.Y., Franciozi C.E.S., Luzo M.V. and Belloti J.C. (2013) Surgical interventions (microfracture, drilling, mosaicplasty and allograft transplantation) for treating isolated cartilage defects of the knee in adults. Cochrane Database of Systematic Reviews, John Wiley & Sons, Ltd 10.1002/14651858.CD010675 PMC6457623 27590275

[21] Murphy C., Mobasheri A., Táncos Z., Kobolák J. and Dinnyés A. (2017) The potency of induced pluripotent stem cells in cartilage regeneration and osteoarthritis treatment. Adv. Exp. Med. Biol. 55–68 10.1007/5584_2017_141 29270885

[22] Xiang X.-N., Zhu S.-Y., He H.-C., Yu X., Xu Y. and He C.-Q. (2022) Mesenchymal stromal cell-based therapy for cartilage regeneration in knee osteoarthritis. Stem Cell Res. Ther. 13, 14 10.1186/s13287-021-02689-9 35012666 PMC8751117

[23] Kafienah W., Mistry S., Dickinson S.C., Sims T.J., Learmonth I. and Hollander A.P. (2006) Three-dimensional cartilage tissue engineering using adult stem cells from osteoarthritis patients. Arthritis Rheumatism 56, 177–187 10.1002/art.22285 17195220

[24] Harrell C.R., Markovic B.S., Fellabaum C., Arsenijevic A. and Volarevic V. (2019) Mesenchymal stem cell-based therapy of osteoarthritis: Current knowledge and future perspectives. Biomedicine Pharmacother. 109, 2318–2326 10.1016/j.biopha.2018.11.099 30551490

[25] Kim C.-H., Lim C.-Y., Lee J.-H., Kim K.C., Ahn J.Y. and Lee E.J. (2018) Human embryonic stem cells-derived mesenchymal stem cells reduce the symptom of psoriasis in imiquimod-induced skin model. Tissue Eng. Regen. Med. 16, 93–102 10.1007/s13770-018-0165-3 30815354 PMC6361099

[26] Gao G., Fan C., Li W., Liang R., Wei C., Chen X. et al. (2021) Mesenchymal stem cells: ideal seeds for treating diseases. Hum. Cell 34, 1585–1600 10.1007/s13577-021-00578-0 34272720 PMC8284686

[27] Takahashi K. and Yamanaka S. (2006) Induction of pluripotent stem cells from mouse embryonic and adult fibroblast cultures by defined factors. Cell 126, 663–676 10.1016/j.cell.2006.07.024 16904174

[28] Yamanaka S. (2020) Pluripotent stem cell-based cell therapy—promise and challenges. Cell Stem Cell 27, 523–531 10.1016/j.stem.2020.09.014 33007237

[29] Castro-Viñuelas R., Sanjurjo-Rodríguez C., Piñeiro-Ramil M., Hermida-Gómez T., Fuentes-Boquete I.M., de Toro-Santos F.J. et al. (2018) Induced pluripotent stem cells for cartilage repair: current status and future perspectives. Eur. Cell Mater. 36, 96–109 10.22203/eCM.v036a08 30204229

[30] Chen M., Jiang Z., Zou X., You X., Cai Z. and Huang J. (2024) Advancements in tissue engineering for articular cartilage regeneration. Heliyon 10, e25400–e25400 10.1016/j.heliyon.2024.e25400 38352769 PMC10862692

[31] Guzzo R.M. and O'Sullivan M.B. (2016) Human pluripotent stem cells: advances in chondrogenic differentiation and articular cartilage regeneration. Curr Mol. Biol. Rep. 2, 113–122 10.1007/s40610-016-0041-7

[32] De Kinderen P., Meester J., Loeys B., Peeters S., Gouze E., Woods S. et al. (2022) Differentiation of induced pluripotent stem cells into chondrocytes: methods and applications for disease modeling and drug discovery. J. Bone Miner. Res. 37, 397–410 10.1002/jbmr.4524 35124831

[33] Saito T., Yano F., Mori D., Kawata M., Hoshi K., Takato T. et al. (2015) Hyaline cartilage formation and tumorigenesis of implanted tissues derived from human induced pluripotent stem cells. Biomed. Res. 36, 179–186 10.2220/biomedres.36.179 26106047

[34] Ko J.-Y., Kim K.-I., Park S. and Im G.-I. (2014) In vitro chondrogenesis and in vivo repair of osteochondral defect with human induced pluripotent stem cells. Biomaterials 35, 3571–3581 10.1016/j.biomaterials.2014.01.009 24462354

[35] Toh W.S., Lee E.H., Guo X.-M., Chan J.K.Y., Yeow C.H., Choo A.B. et al. (2010) Cartilage repair using hyaluronan hydrogel-encapsulated human embryonic stem cell-derived chondrogenic cells. Biomaterials 31, 6968–6980 10.1016/j.biomaterials.2010.05.064 20619789

[36] Lach M., Trzeciak T., Richter M., Pawlicz J. and Suchorska W.M. (2014) Directed differentiation of induced pluripotent stem cells into chondrogenic lineages for articular cartilage treatment. J. Tissue Eng. 5, 2041731414552701–2041731414552701 10.1177/2041731414552701 25383175 PMC4221915

[37] Lach M.S., Kulcenty K., Jankowska K., Trzeciak T., Richter M. and Suchorska W.M. (2018) Effect of cellular mass on chondrogenic differentiation during embryoid body formation. Mol. Med. Rep. 18, 2705–2714 10.3892/mmr.2018.9272 30015965 PMC6102628

[38] Itskovitz-Eldor J., Schuldiner M., Karsenti D., Eden A., Yanuka O., Amit M. et al. (2000) Differentiation of human embryonic stem cells into embryoid bodies comprising the three embryonic germ layers. Mol. Med. 6, 88–95 10.1007/BF03401776 10859025 PMC1949933

[39] Shevde N.K. and Mael A.A. (2012) Techniques in embryoid body formation from human pluripotent stem cells. Basic Cell Culture Protocols 946 535–546 10.1007/978-1-62703-128-8_33 23179854

[40] Nam Y., Rim Y.A., Jung S.M. and Ju J.H. (2017) Cord blood cell-derived iPSCs as a new candidate for chondrogenic differentiation and cartilage regeneration. Stem Cell Res. Ther. 8, 16 10.1186/s13287-017-0477-6 28129782 PMC5273802

[41] Rim Y.A., Nam Y., Park N., Jung H., Lee K., Lee J. et al. (2020) Chondrogenic Differentiation from induced pluripotent stem cells using non-viral minicircle vectors. Cells 9, 582 10.3390/cells9030582 32121522 PMC7140457

[42] Costa M., Cerqueira M.T., Santos T.C., Sampaio-Marques B., Ludovico P., Marques A.P. et al. (2017) Cell sheet engineering using the stromal vascular fraction of adipose tissue as a vascularization strategy. Acta Biomater. 55, 131–143 10.1016/j.actbio.2017.03.034 28347862

[43] Kim M.-J., Son M.J., Son M.-Y., Seol B., Kim J., Park J. et al. (2011) Generation of human induced pluripotent stem cells from osteoarthritis patient-derived synovial cells. Arthritis Rheumatism 63, 3010–3021 10.1002/art.30488 21953087

[44] Augustyniak E., Suchorska W.M., Trzeciak T. and Richter M. (2017) Gene expression profile in human induced pluripotent stem cells: chondrogenic differentiation in vitro, part B. Mol. Med. Rep. 15, 2402–2414 10.3892/mmr.2017.6335 28447733 PMC5428858

[45] Lee J., Taylor S.E.B., Smeriglio P., Lai J., Maloney W.J., Yang F. et al. (2015) Early induction of a prechondrogenic population allows efficient generation of stable chondrocytes from human induced pluripotent stem cells. FASEB J. 29, 3399–3410 10.1096/fj.14-269720 25911615 PMC4511207

[46] Bian L., Guvendiren M., Mauck R.L. and Burdick J.A. (2013) Hydrogels that mimic developmentally relevant matrix and N-cadherin interactions enhance MSC chondrogenesis. Proc. Natl. Acad. Sci. U.S.A. 110, 10117–10122 10.1073/pnas.1214100110 23733927 PMC3690835

[47] Hwang H.S. and Kim H.A. (2015) Chondrocyte apoptosis in the pathogenesis of osteoarthritis. Int. J. Mol. Sci. 16, 26035–26054 10.3390/ijms161125943 26528972 PMC4661802

[48] Li Y., Liu T., Van Halm-Lutterodt N., Chen J., Su Q. and Hai Y. (2016) Reprogramming of blood cells into induced pluripotent stem cells as a new cell source for cartilage repair. Stem Cell Res. Ther. 7, 31 10.1186/s13287-016-0290-7 26883322 PMC4756426

[49] Qu C., Puttonen K.A., Lindeberg H., Ruponen M., Hovatta O., Koistinaho J. et al. (2013) Chondrogenic differentiation of human pluripotent stem cells in chondrocyte co-culture. Int. J. Biochem. Cell Biol. 45, 1802–1812 10.1016/j.biocel.2013.05.029 23735325

[50] Boreström C., Simonsson S., Enochson L., Bigdeli N., Brantsing C., Ellerström C. et al. (2014) Footprint-free human induced pluripotent stem cells from articular cartilage with redifferentiation capacity: a first step toward a clinical-grade cell source. Stem Cells Transl. Med. 3, 433–447 10.5966/sctm.2013-0138 24604283 PMC3973712

[51] Oldershaw R.A., Baxter M.A., Lowe E.T., Bates N., Grady L.M., Soncin F. et al. (2010) Directed differentiation of human embryonic stem cells toward chondrocytes. Nat. Biotechnol. 28, 1187–1194 10.1038/nbt.1683 20967028

[52] Nakagawa Y., Muneta T., Otabe K., Ozeki N., Mizuno M., Udo M. et al. (2016) Cartilage derived from bone marrow mesenchymal stem cells expresses lubricin in vitro and in vivo. PLoS ONE 11, e0148777–e0148777 10.1371/journal.pone.0148777 26867127 PMC4750963

[53] Nejadnik H., Hui J.H., Feng Choong E.P., Tai B.-C. and Lee E.H. (2010) Autologous bone marrow–derived mesenchymal stem cells versus autologous chondrocyte implantation. Am. J. Sports Med. 38, 1110–1116 10.1177/0363546509359067 20392971

[54] Matsumoto Y., Hayashi Y., Schlieve C.R., Ikeya M., Kim H., Nguyen T.D. et al. (2013) Induced pluripotent stem cells from patients with human fibrodysplasia ossificans progressiva show increased mineralization and cartilage formation. Orphanet J. Rare Dis. 8, 190 10.1186/1750-1172-8-190 24321451 PMC3892046

[55] Sui Y., Clarke T. and Singh Khillan J. (2003) Limb bud progenitor cells induce differentiation of pluripotent embryonic stem cells into chondrogenic lineage. Differentiation 71, 578–585 10.1111/j.1432-0436.2003.07109001.x 14686955

[56] Yang W., Lee S., Jo Y.H., Lee K.M., Nemeno J.G., Nam B.M. et al. (2014) Effects of natural cartilaginous extracellular matrix on chondrogenic potential for cartilage cell transplantation. Transplant. Proc. 46, 1247–1250 10.1016/j.transproceed.2013.11.082 24815172

[57] Guzzo R.M., Scanlon V., Sanjay A., Xu R.-H. and Drissi H. (2014) Establishment of human cell type-specific iPS cells with enhanced chondrogenic potential. Stem Cell Rev. Rep. 10, 820–829 10.1007/s12015-014-9538-8 24958240

[58] Urlić I. and Ivković A. (2021) Cell sources for cartilage repair-biological and clinical perspective. Cells 10, 2496 10.3390/cells10092496 34572145 PMC8468484

[59] Langer R. and Vacanti J.P. (1993) Tissue engineering. Science (1979) 260, 920–926 10.1126/science.8493529 8493529

[60] Ovsianikov A., Khademhosseini A. and Mironov V. (2018) The synergy of scaffold-based and scaffold-free tissue engineering strategies. Trends Biotechnol. 36, 348–357 10.1016/j.tibtech.2018.01.005 29475621

[61] Chan B.P. and Leong K.W. (2008) Scaffolding in tissue engineering: general approaches and tissue-specific considerations. Eur. Spine J. 17, 467–479 10.1007/s00586-008-0745-3 19005702 PMC2587658

[62] Sundelacruz S. and Kaplan D.L. (2009) Stem cell- and scaffold-based tissue engineering approaches to osteochondral regenerative medicine. Semin. Cell Dev. Biol. 20, 646–655 10.1016/j.semcdb.2009.03.017 19508851 PMC2737137

[63] O'Brien F.J., Harley B.A., Yannas I.V. and Gibson L.J. (2005) The effect of pore size on cell adhesion in collagen-GAG scaffolds. Biomaterials 26, 433–441 10.1016/j.biomaterials.2004.02.052 15275817

[64] Wei G. and Ma P.X. (2004) Structure and properties of nano-hydroxyapatite/polymer composite scaffolds for bone tissue engineering. Biomaterials 25, 4749–4757 10.1016/j.biomaterials.2003.12.005 15120521

[65] De Pieri A., Rochev Y. and Zeugolis D.I. (2021) Scaffold-free cell-based tissue engineering therapies: advances, shortfalls and forecast. NPJ Regen. Med. 6, 18 10.1038/s41536-021-00133-3 33782415 PMC8007731

[66] Harley W., Yoshie H. and Gentile C. (2021) Three-dimensional bioprinting for tissue engineering and regenerative medicine in down under: 2020 australian workshop summary. ASAIO. J. 67, 363–369 10.1097/MAT.0000000000001389 33741790

[67] Jain P., Kathuria H. and Dubey N. (2022) Advances in 3D bioprinting of tissues/organs for regenerative medicine and in-vitro models. Biomaterials 287, 121639 10.1016/j.biomaterials.2022.121639 35779481

[68] Xiongfa J., Hao Z., Liming Z. and Jun X. (2018) Recent advances in 3D bioprinting for the regeneration of functional cartilage. Regenerative Med. 13, 73–87 10.2217/rme-2017-0106 29350587

[69] Agarwal S., Saha S., Balla V.K., Pal A., Barui A. and Bodhak S. (2020) Current developments in 3D bioprinting for tissue and organ regeneration–a review. Front Mech. Eng. 6, 1–22 10.3389/fmech.2020.589171

[70] Murphy S.V. and Atala A. (2014) 3D bioprinting of tissues and organs. Nat. Biotechnol. 32, 773–785 10.1038/nbt.2958 25093879

[71] Gu Z., Fu J., Lin H. and He Y. (2020) Development of 3D bioprinting: from printing methods to biomedical applications. Asian J. Pharm. Sci. 15, 529–557 10.1016/j.ajps.2019.11.003 33193859 PMC7610207

[72] Nguyen D., Hägg D.A., Forsman A., Ekholm J., Nimkingratana P., Brantsing C. et al. (2017) Cartilage tissue engineering by the 3D bioprinting of iPS cells in a nanocellulose/alginate bioink. Sci. Rep. 7, 658 10.1038/s41598-017-00690-y 28386058 PMC5428803

[73] Yang X., Lu Z., Wu H., Li W., Zheng L. and Zhao J. (2018) Collagen-alginate as bioink for three-dimensional (3D) cell printing based cartilage tissue engineering. Mater. Sci. Eng. C 83, 195–201 10.1016/j.msec.2017.09.002 29208279

[74] Hontani K., Onodera T., Terashima M., Momma D., Matsuoka M., Baba R. et al. (2019) Chondrogenic differentiation of mouse induced pluripotent stem cells using the three‐dimensional culture with ultra‐purified alginate gel. J. Biomed. Mater. Res. A 107, 1086–1093 10.1002/jbm.a.36615 30665260

[75] Shukla A.K., Gao G. and Kim B.S. (2022) Applications of 3D bioprinting technology in induced pluripotent stem cells-based tissue engineering. Micromachines (Basel) 13, 155 10.3390/mi13020155 35208280 PMC8876961

[76] Gatenholm P., Martinez H., Karabulut E., Amoroso M., Kölby L., Markstedt K. et al. (2016) Development of nanocellulose-based bioinks for 3D bioprinting of soft tissue. 3D Print. Biofabr. 1–23 10.1007/978-3-319-40498-1_14-1

[77] Lee A., Hudson A.R., Shiwarski D.J., Tashman J.W., Hinton T.J., Yerneni S. et al. (2019) 3D bioprinting of collagen to rebuild components of the human heart. Science (1979) 365, 482–487 10.1126/science.aav9051 31371612

[78] Zhang Y., Mu Y., He Y., Li Z., Mi G., Liu Y. et al. (2021) Upregulated expression of transforming growth factor-β receptor I/II in an endemic Osteoarthropathy in China. BMC Musculoskelet. Disord. 22, 1051 10.1186/s12891-021-04939-6 34930205 PMC8690967

[79] Ye K., Felimban R., Traianedes K., Moulton S.E., Wallace G.G., Chung J. et al. (2014) Chondrogenesis of infrapatellar fat pad derived adipose stem cells in 3D printed chitosan scaffold. PLoS ONE 9, e99410–e99410 10.1371/journal.pone.0099410 24918443 PMC4053433

[80] Sun Y., You Y., Jiang W., Wang B., Wu Q. and Dai K. (2020) 3D bioprinting dual-factor releasing and gradient-structured constructs ready to implant for anisotropic cartilage regeneration. Sci. Adv. 6, 10.1126/sciadv.aay1422 PMC11206535 32917692

[81] Daly A.C. and Kelly D.J. (2019) Biofabrication of spatially organised tissues by directing the growth of cellular spheroids within 3D printed polymeric microchambers. Biomaterials 197, 194–206 10.1016/j.biomaterials.2018.12.028 30660995

[82] He Y., Derakhshanfar S., Zhong W., Li B., Lu F., Xing M. et al. (2020) Characterization and application of carboxymethyl chitosan-based bioink in cartilage tissue engineering. J. Nanomater 2020, 1–11 10.1155/2020/2057097

[83] Sadeghianmaryan A., Naghieh S., Alizadeh Sardroud H., Yazdanpanah Z., Afzal Soltani Y., Sernaglia J. et al. (2020) Extrusion-based printing of chitosan scaffolds and their in vitro characterization for cartilage tissue engineering. Int. J. Biol. Macromol. 164, 3179–3192 10.1016/j.ijbiomac.2020.08.180 32853616

[84] Deng C., Yang J., He H., Ma Z., Wang W., Zhang Y. et al. (2021) 3D bio-printed biphasic scaffolds with dual modification of silk fibroin for the integrated repair of osteochondral defects. Biomater Sci. 9, 4891–4903 10.1039/D1BM00535A 34047307

[85] Keriquel V., Oliveira H., Rémy M., Ziane S., Delmond S., Rousseau B. et al. (2017) In situ printing of mesenchymal stromal cells, by laser-assisted bioprinting, for in vivo bone regeneration applications. Sci. Rep. 7, 1778 10.1038/s41598-017-01914-x 28496103 PMC5431768

